# Case report: Ruptured internal carotid artery fusiform aneurysm mimicking pituitary apoplexy after stereotactic radiosurgery

**DOI:** 10.3389/fneur.2023.1219372

**Published:** 2023-08-04

**Authors:** Peng Wei Wang, Ming Hsuan Chung, Shao Wei Feng, Hsiang Chih Liao, Yi Chieh Wu, Dueng Yuan Hueng, Yun Ju Yang, Da Tong Ju

**Affiliations:** ^1^Department of Surgery, Taoyuan Armed Forces General Hospital, Taoyuan, Taiwan; ^2^Department of Neurological Surgery, Tri-Service General Hospital and National Defense Medical Center, Taipei, Taiwan

**Keywords:** fusiform aneurysm, radiosurgery (SRS), pituitary tumor, apoplexy, internal carotid aneurysm

## Abstract

Pituitary adenomas are benign tumors of the anterior pituitary gland for which surgery or pharmacological treatment is the primary treatment. When initial treatment fails, radiation therapy should be considered. There are several case reports demonstrating radiation-induced vascular injury. We report an adult patient who presented with headache and diplopia for 6 months and a sellar tumor with optic chiasm compression. The patient received transnasal surgery, and the tumor was partially removed, which demonstrated adenoma. Stereotactic radiosurgery (SRS) was arranged. However, owing to progressive tumor growth, the patient received further transnasal surgery and stereotactic radiosurgery (SRS). After 14 years, the patient reported the sudden onset of headache and diplopia, and a ruptured fusiform aneurysm from the left internal carotid artery with pituitary apoplexy was diagnosed. The patient received transarterial embolization of the aneurysm. There were no complications after embolization, and this patient was ambulatory on discharge with blindness in the left eye and cranial nerve palsies. Aneurysm formation may be a complication of SRS, and it may occur after several years. Further research is needed to investigate the pathogenesis of radiosurgery and the development of cerebral aneurysms.

## Case description

**Figure 1:**
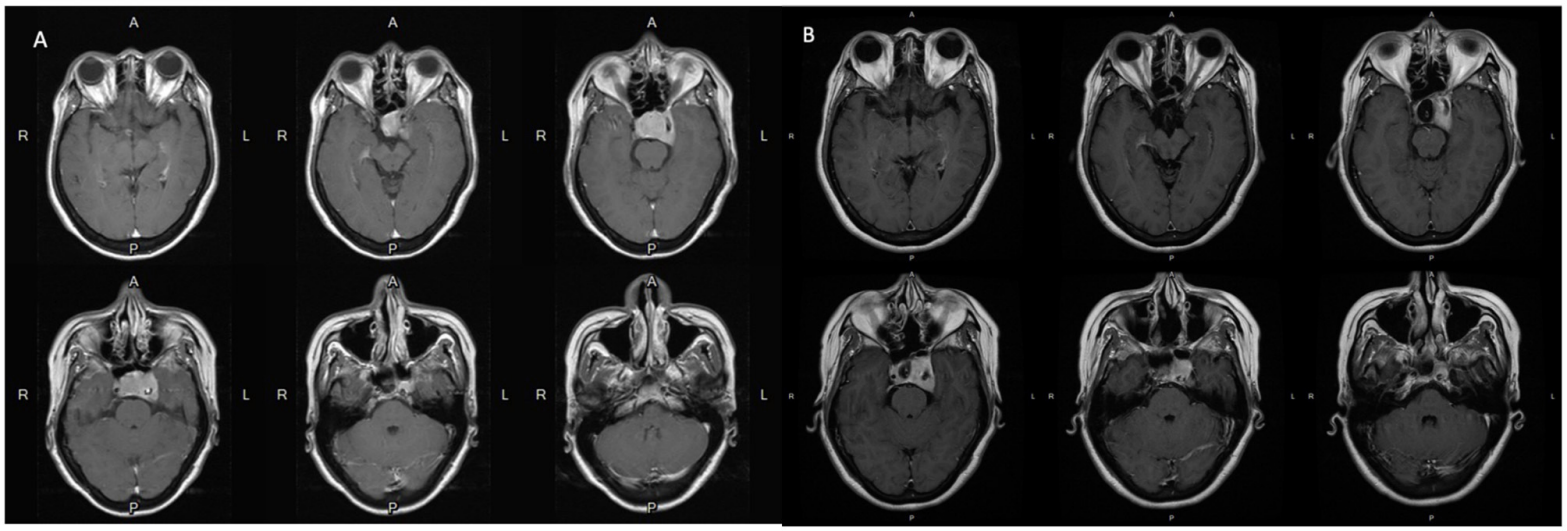
**(A)** Pre-operative MRI scans with contrast show the pituitary tumor measured 3.6 cm x 2.35 cm x 2.83 cm causes erosion of the sellar floor and encasement of the left cavernous structure including the internal carotid artery. **(B)** Post-operative MRI scans with contrast show that the residual tumor is still located mainly in the left side of the sellar fossa and parasellar structures including the cavernous sinus and Meckel's cave.

**Figure 2:**
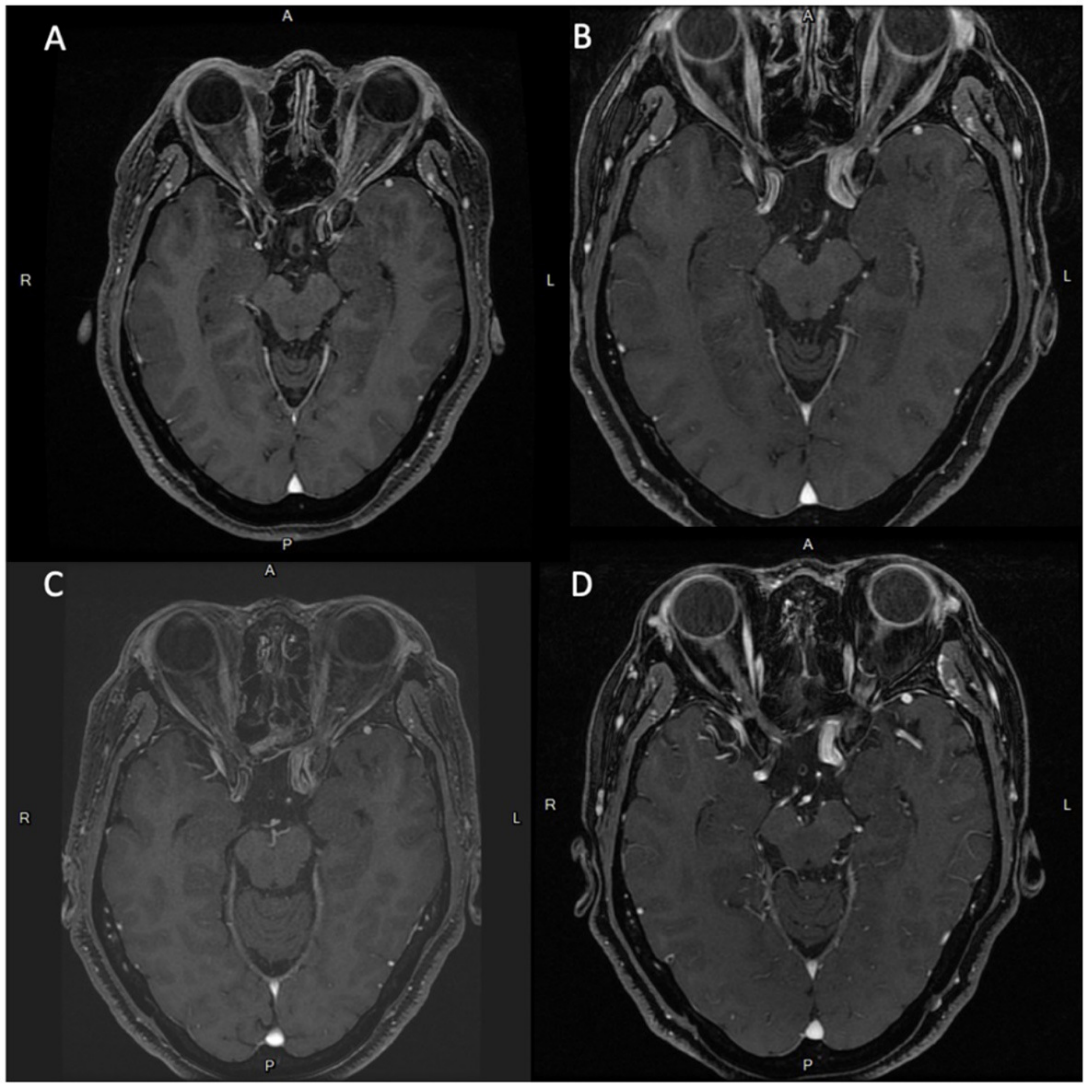
Series of brain MRI scans with contrast shows newly noted focal dilation of left cavernous ICA (diameter: 1.3 cm) and medial protrusion (0.75 cm) in the cavernous ICA. since 2020.06. **(A)** Brain MRI scans in 2008.10. **(B)** Brain MRI scans in 2018.01. **(C)** Brain MRI scans in 2019.06. **(D)** Brain MRI scans in 2020.06.

An adult patient in their 40's reported progressive headaches, vision loss in the left eye, and diplopia for 6 months. Magnetic resonance imaging (MRI) revealed a sellar tumor with parasellar and suprasellar extension and optic chiasm compression (Figure 1A). No endocrine abnormalities were noted, and neurosurgical treatment was suggested owing to the development of neurological symptoms. This patient underwent transnasal transsphenoidal surgery, and the sellar tumor was partially removed (Figure 1B). The surgical pathology report showed adenoma, and fractionated radiotherapy with 4,500 cGy that was divided to the anterior, left, and right parts of the tumor in 24 sessions was performed. However, progressive tumor enlargement was seen in follow-up imaging studies, and headache had developed. This patient underwent further transnasal transsphenoidal surgery with debulking of the tumor, and stereotactic radiosurgery (X-knife) with 1,200 cGy delivering 90% isodose volume that covered 98% of tumor volume was performed. An annual follow-up image demonstrated no interval change in tumor size and no vascular abnormalities (Figures 2A–D). However, 14 years after SRS, the patient was sent to our emergency department due to the sudden onset of headache, dizziness, diplopia, and blindness in the left eye for 1 day. In the emergency department, a loss of pupillary light reflex in the left eye, and oculomotor nerve and abducens nerve palsies were found.

## Diagnostic assessment

**Figure 3:**
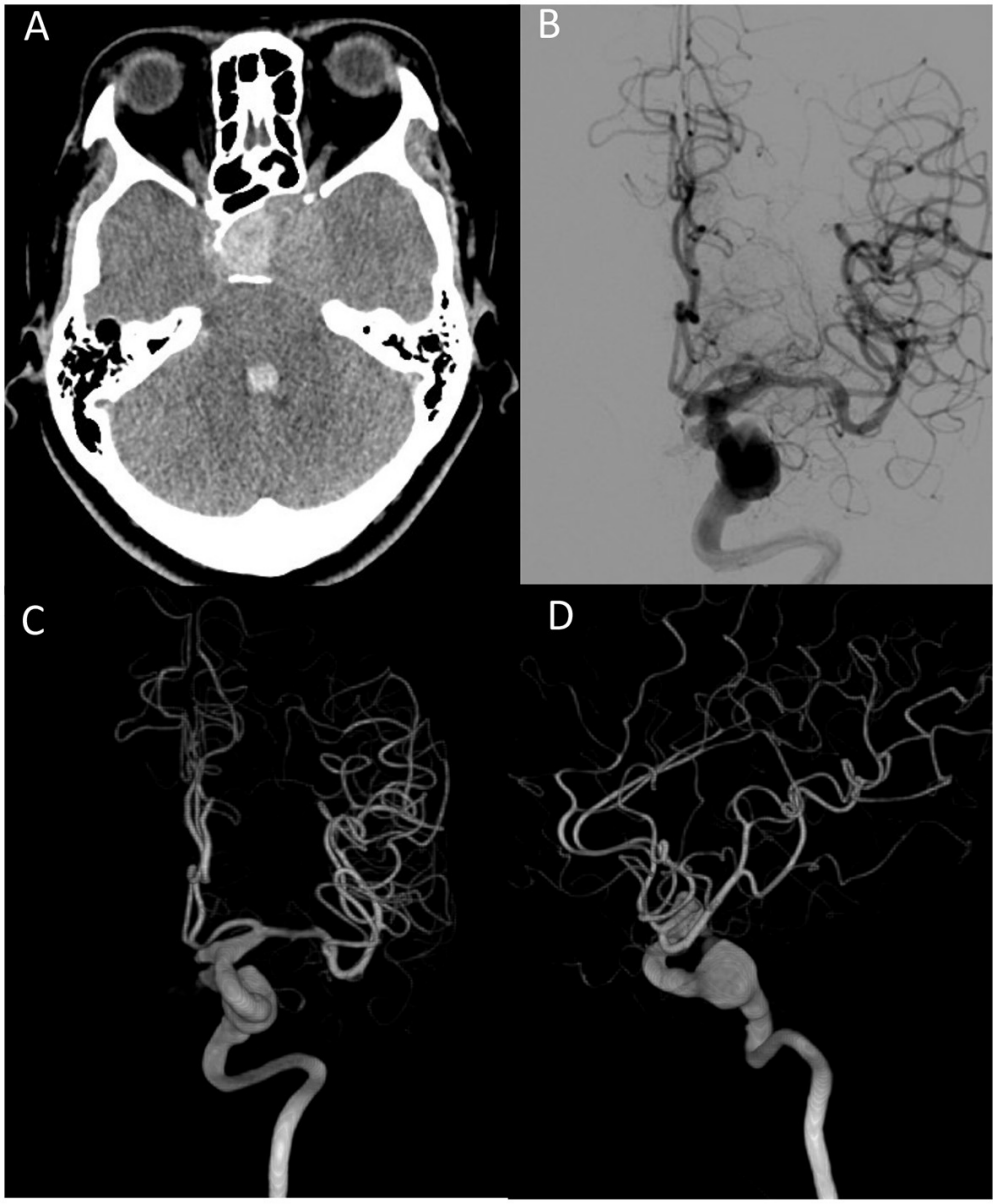
**(A)** Brain CT scan shows hemorrhage at the sellar turcica and intraventricular hemorrhage. **(B)** Fusiform aneurysm from the left ICA. Arrow indicates contrast extravasation. **(C)** Anterior–posterior view of 3-D reconstruction angiography. **(D)** Lateral view of 3D reconstruction angiography. ICA, internal carotid artery.

A hematoma at the sellar turcica and an intraventricular hemorrhage were found on brain computer tomography (CT) (Figure 3A). A cerebral angiogram revealed a fusiform aneurysm measuring 1.55 x 1.45 cm arising from the cavernous segment of the left internal carotid artery, with contrast extravasation from the aneurysm to the sellar turcica, and another saccular aneurysm measuring 0.7 x 0.6 cm arising from the left posterior communicating artery (Figures 3B–D). Because of the location (cavernous segment of the ICA) and shape of the fusiform aneurysm, it was difficult to approach and perform clipping or bypass surgery. This patient was treated with stent-assisted coiling embolization of the ICA aneurysm. A self-expandable stent was deployed spanning the left distal internal carotid artery at the C4 segment, covering the aneurysm orifice. A total of 32 coils were delivered into the aneurysm. There were no complications after embolization, and the patient was ambulatory on discharge with blindness in the left eye and cranial nerve palsies.

## Discussion

SRS is used for several types of intracranial tumors. In 1968, Dr. Leksell treated the first pituitary macroadenoma with SRS, and many thousands of patients with pituitary adenomas have since been treated with SRS. The radiation effect stabilizes tumor growth with an average control rate of 68–100% and normalizes hormone levels. However, complications following radiosurgery for pituitary adenoma have been reported, including hypopituitarism, cranial nerve injury, adjacent vascular structure injury, and brain parenchyma injury. Injury to the cavernous segment of the internal carotid artery is rare, and only two cases of symptomatic carotid artery stenosis have been reported.

The definition of radiation-induced aneurysms is still under debate due to the absence of pathognomonic radiographic or histologic features. As a result, a more appropriate description would be aneurysms occurring in an irradiation field. Five cases of aneurysm formation after SRS for the treatment of intracranial tumors have been reported, four of which were acoustic neuromas and one was bilateral retinoblastoma (1). Ruptured anterior inferior cerebellar artery aneurysms occurred in the four patients with acoustic neuromas, and an anterior cerebral artery aneurysm occurred in the patient with retinoblastoma. Four of the five cases (80%) were alive after treatment, and the time between irradiation and the discovery of an aneurysm ranged from 6 to 11 years.

The pathophysiology and histologic changes in radiation-induced aneurysms are not well understood. A possible explanation is that ionizing radiation causes cell death, leading to endothelial cell dysfunction, vasa vasorum injury, and accelerated atherosclerosis in acute and chronic time periods. This then resulted in intimal and adventitia injury of the artery, which Barletta et al. suggested are associated with fusiform aneurysm development (2). In addition, radiation can also induce inflammatory cytokine cascades, which persist for years (3). Chalouhi et al. (4) reported that inflammatory reactions associated with intracranial aneurysm formation begin with endothelial dysfunction, followed by the production of cytokines such as interleukin, tumor necrosis factor-alpha, matrix metalloproteinases, and prostaglandin E2. These inflammatory responses result in the disruption of the internal elastic lamina of vessels, vascular smooth muscle cell apoptosis, and the development of an aneurysm. Direct cell death and inflammatory responses after radiation may cause vessel wall injury and subsequent aneurysm formation.

Moreover, the incidence of intracranial aneurysms in patients with pituitary adenoma is 2.3–7.4% higher than in the general population and in patients with other brain tumors (5). However, apoplexy from a ruptured cerebral aneurysm is rare. Five cases in the literature reported the coexistence of ruptured aneurysms (5), located at the anterior cerebral artery and posterior communicating artery. Local circulatory stress, endocrinological effect, mechanical effect, and direct invasion have been proposed as possible mechanisms of aneurysm formation in patients with pituitary adenoma (5). However, our case is different from previous cases due to the unique location of the aneurysm, the absence of an endocrinological effect, and the fact that the aneurysm developed over a long time period. This time period reinforces that SRS was most likely the cause of the aneurysm rather than the adenoma itself.

The treatment of radiation-induced fusiform cerebral aneurysms is not well-established. Endovascular treatment with or without bypass procedures should be considered in these cases. However, several issues should be considered before treatment. First, the fragile radiation-induced aneurysm wall is prone to rupture, so the possibility of rupture during embolization should be considered. Second, stenosis of the internal carotid artery, middle cerebral artery, and anterior cerebral artery due to radiation may increase the risk of embolic events during catheter passage. Careful instrument manipulation with dual antiplatelet therapy and heparization is needed to prevent infarction events. Moreover, a regional radiation effect may increase the risk of recanalization and new aneurysm formation. Hence, these patients should be closely followed after the procedure.

## Patient perspective

The actual pathogenesis of aneurysm formation after SRS is still under debate. The duration of aneurysmal formation following SRS to treat intracranial tumors can be several years. How long is the recommended follow-up after radiotherapy?

The fragile radiation-induced aneurysm wall is prone to rupture, so the possibility of rupture during embolization should be considered.

Patients with pituitary adenoma are at greater risk of aneurysm development. Due to the effect of radiation on cerebral vessels, the risk of aneurysm should be explained and well discussed before treatment.

## Data availability statement

The original contributions presented in the study are included in the article/supplementary material, further inquiries can be directed to the corresponding author.

## Ethics statement

The studies involving human participants were reviewed and approved by the Tri-Service General Hospital Institutional Review Board (Approval No. A202215217). The patients/participants provided their written informed consent to participate in this study. Written informed consent was obtained from the individual(s) for the publication of any potentially identifiable images or data included in this article.

## Author contributions

PW, MC, and DJ were responsible for the study conceptualization. HL, YW, and SF were responsible for the study procedures. PW, YY, and DH analyzed and interpreted the study data. PW and DJ were the major contributors in writing the original draft. PW, MC, YY, DH, and DJ reviewed and edited the manuscript. All authors read and approved the final manuscript.
