# Corrigendum: Case report: Ruptured internal carotid artery fusiform aneurysm mimicking pituitary apoplexy after stereotactic radiosurgery

**DOI:** 10.3389/fneur.2023.1301436

**Published:** 2023-10-10

**Authors:** Peng Wei Wang, Ming Hsuan Chung, Shao Wei Feng, Hsiang Chih Liao, Yi Chieh Wu, Dueng Yuan Hueng, Yun Ju Yang, Da Tong Ju

**Affiliations:** ^1^Department of Surgery, Taoyuan Armed Forces General Hospital, Taoyuan, Taiwan; ^2^Department of Neurological Surgery, Tri-Service General Hospital and National Defense Medical Center, Taipei, Taiwan

**Keywords:** fusiform aneurysm, radiosurgery (SRS), pituitary tumor, apoplexy, internal carotid aneurysm

In the published article, there was an error in affiliation 2. Instead of “Department of Neurological Surgery, Tri-Service General Hospital, Taipei, Taiwan”, it should be “Department of Neurological Surgery, Tri-Service General Hospital and National Defense Medical Center, Taipei, Taiwan”.

The authors apologize for this error and state that this does not change the scientific conclusions of the article in any way. The original article has been updated.

